# Supplementary material for: Local Injection of Stem Cells Can Be a Potential Strategy to Improve Bladder Dysfunction after Outlet Obstruction in Rats
Source: Int J Mol Sci. 2024 Jul 30;25(15):8310. doi: 10.3390/ijms25158310 (PMC11313184; doi:10.3390/ijms25158310)
Supplement: Supplementary file 1 [file ijms-25-08310-s001.zip › Supplementary Table 1. Western blot primary antibody.pdf]

Supplementary Table S1. Primary antibodies used for the western blot investigations.

Western blot primary antibody	Type, Catalogue number	Isotype	Dilution	Host species	Manufacturer
Connexin 43	polyclonal, ab11370	IgG	1:2000	Rabbit	Abcam, Cambridge, USA
Smad2/3	monoclonal, ab202445	IgG	1:1000	Rabbit	Abcam, Cambridge, USA
Caveolin 1	monoclonal, sc-53564	IgG	1:500	Mouse	Santa Cruz Biotechnology, USA
Caveolin 3	polyclonal, ab2912	IgG	1:1000	Rabbit	Abcam, Cambridge, USA
Collagen 1	monoclonal, ab6308	IgG1	1:1000	Mouse	Abcam, Cambridge, USA
Collagen 3	monoclonal, ab6310	IgG1	1:1000	Mouse	Abcam, Cambridge, USA
TGF-β1	monoclonal, sc-130348	IgG	1:500	Mouse	Santa Cruz Biotechnology, USA
β-actin	monoclonal, #3700S	IgG	1:3000	Mouse	Cell Signaling Technology, Danvers, MA
