# Supplementary material for: Local Injection of Stem Cells Can Be a Potential Strategy to Improve Bladder Dysfunction after Outlet Obstruction in Rats
Source: Int J Mol Sci. 2024 Jul 30;25(15):8310. doi: 10.3390/ijms25158310 (PMC11313184; doi:10.3390/ijms25158310)
Supplement: Supplementary file 1 [file ijms-25-08310-s001.zip › Supplementary Table 2. Immunofluorescence primary antibody.pdf]

Supplementary Table S2. Primary antibodies used for the immunofluorescence investigations.

Immunofluorescence primary antibody	Type, Catalogue number	Isotype	Dilution	Host species	Manufacturer
α-SMA	monoclonal, A2547	IgG	1:100000	Mouse	Sigma, St. Louis, MO, USA
MYH11	monoclonal, sc-6956	IgG	1:100	Mouse	Santa Cruz Biotechnology, USA
PKG	polyclonal, ADI-KAP-PK005	IgG	1:200	Rabbit	Enzo Life Sciences, Exeter, UK
M2	polyclonal, AB5166	IgG	1:500	Rabbit	MilliporeSigma, Rockville, MD, USA
M3	polyclonal, sc-9108	IgG	1:250	Rabbit	Santa Cruz Biotechnology, USA
P2X1	polyclonal, sc-25692	IgG	1:250	Rabbit	Santa Cruz Biotechnology, USA
NK2	polyclonal, bs-0123R	IgG	1:100	Rabbit	Bioss, Beijing, China
